# Is the effect of precipitation on acute gastrointestinal illness in southwestern Uganda different between Indigenous and non-Indigenous communities?

**DOI:** 10.1371/journal.pone.0214116

**Published:** 2019-05-02

**Authors:** Johanna Busch, Lea Berrang-Ford, Sierra Clark, Kaitlin Patterson, Emma Windfeld, Blanaid Donnelly, Shuaib Lwasa, Didacus Namanya, Sherilee L. Harper, for the IHACC team

**Affiliations:** 1 Department of Geography, McGill University, Montreal, Canada; 2 Priestley International Centre for Climate, University of Leeds, Leeds, United Kingdom; 3 Department of Epidemiology, Biostatistics, and Occupational Health, McGill University, Montreal, Canada; 4 Department of Population Medicine, University of Guelph, Guelph, Canada; 5 Community Veterinary Outreach, Ottawa, Canada; 6 Department of Geography, Makerere University, Kampala, Uganda; 7 Ministry of Health, Kampala, Uganda; 8 School of Public Health, University of Alberta, Edmonton, Canada; New Jersey Department of Health, UNITED STATES

## Abstract

Acute gastrointestinal illness (AGI) is a global public health priority that often disproportionately effects Indigenous populations. While previous research examines the association between meteorological conditions and AGI, little is known about how socio-cultural factors may modify this relationship. This present study seeks to address this research gap by comparing AGI prevalence and determinants between an Indigenous and non-Indigenous population in Uganda. We estimate the 14-day self-reported prevalence of AGI among adults in an Indigenous Batwa population and their non-Indigenous neighbours using cross-sectional panel data collected over four periods spanning typically rainy and dry seasons (January 2013 to April 2014). The independent associations between Indigenous status, precipitation, and AGI are examined with multivariable multi-level logistic regression models, controlling for relative wealth status and clustering at the community level. Estimated prevalence of AGI among the Indigenous Batwa was greater than among the non-Indigenous Bakiga. Our models indicate that both Indigenous identity and decreased levels of precipitation in the weeks preceding the survey period were significantly associated with increased AGI, after adjusting for confounders. Multivariable models stratified by Indigenous identity suggest that Indigenous identity may not modify the association between precipitation and AGI in this context. Our results suggest that short-term changes in precipitation affect both Indigenous and non-Indigenous populations similarly, though from different baseline AGI prevalences, maintaining rather than exacerbating this socially patterned health disparity. In the context of climate change, these results may challenge the assumption that changing weather patterns will necessarily exacerbate existing socially patterned health disparities.

## Introduction

Global climate change is expected to have substantial impacts on human health [1, 2]. Sub-Saharan Africa may be particularly vulnerable as meteorological changes are projected to be severe and resources for adaptive capacity are lacking [3, 4]. Acute gastrointestinal illness (AGI), a climate-sensitive health outcome, is a leading cause of morbidity and child mortality worldwide and in Africa [5, 6]. In the tropics and subtropics, the burden of AGI is expected to increase as temperature and precipitation vary due to climate change, leading to decreased water quality and availability [7, 8]. A systematic review of global weather and waterborne disease trends identified positive associations between increased rates of diarrheal disease and heavy rainfall events [7]; while in Africa, several studies have found the opposite association, including increased diarrheal disease occurrence during periods of low precipitation [9–11]. Large uncertainties still exist in these associations reflecting a paucity of research examining how meteorological conditions with AGI manifests differently in diverse environmental and socio-economic contexts [8, 12]. More contextually focused research is needed to examine how the precipitation-AGI relationship is influenced by socio-demographic and environmental variables.

Contextual understanding is particularly important because the AGI burden, like many other climate-sensitive health outcomes, is not homogenously distributed along spatial and social gradients. Some groups, such as Indigenous peoples, may currently experience greater risk of AGI than others. Indigenous populations, worldwide and in Africa, often experience poorer health outcomes than their non-Indigenous counterparts [13, 14]. Poverty, discrimination, limited access to health care, hygiene conditions, and loss of traditional lands are often at the root of disparities in Indigenous health outcomes [15, 16]. In terms of AGI, research from Brazil and Canada indicate that Indigenous populations may experience higher rates of AGI than non-Indigenous populations [17, 18].

Climatic and socio-demographic factors are typically considered independently. Thus, it is unclear how these factors interact. It is often assumed that climate change will exacerbate health disparities along existing social gradients (i.e., currently vulnerable groups will become even more vulnerable) [19]; however, the health impacts of climate change may also maintain, rather than exacerbate, current health disparities between different groups, or even create completely different gradients of health [20]. Understanding how climate change will affect existing health inequalities is crucial to reducing disease burden. Quantitative research on how climate change will affect existing gradients of climate sensitive health outcomes, such as AGI, is lacking, especially for Indigenous populations. This paper sought to begin filling this research gap by comparing AGI prevalence and determinants between an Indigenous and non-Indigenous population in Uganda. This study (1) estimated AGI prevalence in an Indigenous and neighbouring non-Indigenous population, (2) examined the association between Indigenous identity and AGI, and (3) assessed how Indigenous identity might modify the effect of precipitation on AGI.

## Methods

### Study population

Kanungu District in southwestern Uganda (1,274 km^2^) has an estimated total population of 250,000 people, including the non-Indigenous majority, the Bakiga (> 99% of the total population), who rely on subsistence farming, cash cropping (e.g. tea and coffee), small-scale livestock holdings, and/or tourism from Bwindi Impenetrable National Park (BINP) as sources of income [21, 22]. The Indigenous Batwa are a minority population (< 1% of the total population) [21, 23] who traditionally lived as hunter-gatherers in the forest until they were evicted from their ancestral land without compensation in 1991 due to the creation of BINP [24]. Since then, the Batwa have settled in camps outside of the park and are transitioning to agricultural livelihoods [25, 26]. This transition has had very limited success, and the Batwa remain one of the most impoverished groups in Uganda, consistently reporting poorer health outcomes than their non-Indigenous Bakiga neighbors, including higher infant mortality rates [14, 27], higher malaria prevalence, and intestinal parasitic loads [28], as well as less access to prevention measures (i.e., insecticidal mosquito nets) [29].

The Batwa also experience a high prevalence of AGI relative to other international prevalence estimates [30]. Significant vulnerability to existing and future climate sensitive health risks has been identified in both Batwa [23] and Bakiga communities [31]. Notably, AGI has been identified as a priority health concern by both the Batwa and Bakiga [23, 30, 31].

### Study design and sample

Cross-sectional face-to-face surveys were administered four times (January 2013; July 2013; January 2014; and April 2014) in each of the 10 Batwa settlements in Kanungu District. Within this region, April is typically a high precipitation month, July is low a low precipitation month, and January is moderate. Of the small total population of Batwa in Kanungu District, a census of all Batwa adults present (18 years in July 2013) was attempted during all four survey periods. During two of these survey periods (July 2013 and April 2014) the Batwa’s non-Indigenous neighbors, the Bakiga, were also surveyed. A two-step proportional systematic random-sample of Bakiga households in the 10 local councils (LC) that contain Batwa settlements was conducted to achieve a 40% random sample of adults.

### Data collection

For each of the four survey periods, a questionnaire was administered to participants in Rukiga, the local language, by trained local surveyors from Kanungu District. The questionnaire consisted of two sections: (1) an individual-level health questionnaire and (2) a household-level questionnaire. The individual-level questionnaires collected demographic information, self- reported occurrence of diarrhea and/or vomiting in the previous 14 days, related symptoms, and health-seeking behavior. The household questionnaire collected information on water sources, sanitation and hygiene practices, ownership and exposure to animals, and socioeconomic indicators [30].

### AGI case definition

The AGI case definition used for this study was the presence of any self-reported symptoms of vomiting or diarrhea in the 14 days before the survey date, excluding participants who self-reported during the survey that their symptoms were due to chronic gastrointestinal illness, pregnancy, or the use of medication and/or alcohol/ drugs [30]. A 14-day recall period was determined as an appropriate and reliable recall period following pilot research and consultation with local partners [30].

### Precipitation data

Participants’ precipitation exposure was determined using estimated daily rainfall in the weeks prior to their individual survey dates. The *Rainfall Estimator*, version 2.0 (RFE2), algorithm [32] was used to estimate daily rainfall for the study region. This algorithm corrects total daily precipitation estimates (mm) from high resolution infrared satellite images using ground rain gauge data and satellite microwave measurements in a calibration equation [33]. The corrected daily precipitation estimates were interpolated to the study region. Daily precipitation estimates were validated using local meteorological measurements from a weather station in Buhoma, which is a central community in the study region. These precipitation estimates were highly correlated with the station measurements with a confidence interval exceeding 99% confidence level [32]. For each survey period, we summed the total precipitation (mm) within each week for the 12 weeks leading up to each survey period, and then linked the weekly precipitation totals to each participant by survey date.

### Statistical analysis

First, the 14-day adult prevalence of AGI among the Batwa and Bakiga was calculated for each survey period by dividing the number of cases meeting our AGI case definition by the total number of adult respondents. We also calculated 95% confidence intervals around each prevalence estimate.

The potential association of AGI with Indigenous identity and precipitation was modeled using multi-level multivariable logistic regression. Indigenous identity and precipitation exposure (log total precipitation in the 2 to 4 weeks before the survey) [7] were included in the model as independent variables. Total weekly precipitation was log transformed because of its skewed distribution. Five different precipitation accumulation periods were considered in our model (2 to 4, 2 to 5, 2 to 6, 2 to 7, and 2 to 8 weeks prior to the survey) with similar results. We used the total two-week precipitation in the 2 to 4 weeks before each survey date (i.e., between 15 and 29 days prior to each survey) in the final analysis due to the delayed response relationship between precipitation and AGI [10]. Considering our AGI recall period covered the 1 to 14 days prior to each survey, we omitted these periods of precipitation exposure from the analysis so as to not attribute the exposure events after the disease onset.

We determined *a priori* to control for wealth using a household asset-based wealth index calculated using principal component analysis (PCA). Variables included in the PCA were based on a previous study in the same context [28] and included cell phone, radio and bicycle ownership, and receiving remittances [34]. A dummy variable was created reflecting those above and below the median level of wealth. Additional independent variables that were considered for inclusion in the model as potential confounders were education (none, some primary, and completed primary or above), sex, employment, availability of household washing facilities and soap, toilet type, water source, water treatment, and ownership and exposure to animals. Variables related to hygiene, water safety, and exposure to animals were considered as independent variables in the model as these are known determinants of AGI [35].

To build our multi-level multivariable logistic regression model, first the unconditional associations between potential confounding variables and the outcome (AGI occurrence) were explored in univariable logistic regression models (AGI occurrence and one other variable). Potential confounders with a p-value < 0.25 were considered for inclusion as controls in the full model [36]. Collinearity between all independent variables was assessed using Spearman’s rank correlation coefficients with a cut-off of |0.7|. Through a manual backwards elimination model building process (iteratively removing independent variables from the full model), independent variables that were not identified as confounders (i.e., coefficients changing more than 25%), and were not significant (<0.1 p-value) were removed from the full model [36].

A series of multi-level models that would control for the potential latent effects on AGI occurrence operating within-community or within-individuals over time (across survey periods) were tested. Separately, a standard logistic model, a within-individual random effects model (random intercept for individuals), and a within-community random effects model were examined. Both random effects models assumed an exchangeable correlation structure within individuals over time or among individuals within communities. We chose the within-community random effects model based on the structure of our data and comparison of Bayesian Information Criterion (BIC) values of model fit [37, 38].

To identify whether Indigenous identity modified the effect of precipitation on AGI prevalence, we stratified our multivariable mixed-effects logistic regression model by community Indigeneity. Wealth and within-community effects were controlled in the stratified models. The associations between precipitation and odds of AGI, as well as the model predicted probabilities of AGI, were compared between the stratified models to determine whether precipitation affects AGI occurrence differently or similarly in the Indigenous and non-Indigenous groups. All data analyses were conducted using Stata v. 13 (Stata Corp., USA).

### Research ethics

This study was approved by the Research Ethics Boards at McGill University and the University of Guelph and is consistent with the Canadian Tri-Council’s policies and requirements for the Ethical Conduct of Research Involving Human Subjects. National research ethics approval mechanisms in Uganda were not active from approximately 2011–2015, covering the period of data collection for this study. We were thus unable to acquire formal Ugandan national approval for the data collected and presented here. Informed oral consent was obtained from all participants. This study is a part of the Indigenous Health Adaptation to Climate Change (IHACC) project, a larger international initiative with parallel research in the Canadian Arctic and the Peruvian Amazon (www.ihacc.ca).

## Results

A total of 1,776 individual questionnaires were completed by adults across all four survey periods, of which 55.3% (982) were Batwa and 44.7% (794) were Bakiga. Response rates among the Batwa range from 93% (July 2013) to 99% (January 2013). Response rates among the Bakiga were 99% (July 2013) and 95% (April 2014). Among both groups, more women than men were surveyed (62.6% compared with 37.3%). Men may have been less likely to participate because they were looking for work or actively working outside of the community during the survey periods. Batwa adults were slightly younger (mean age 36.3 years) than Bakiga adults (mean age 38.3 years). According to household-level self-reported surveys, fewer Bakiga individuals (46.2%) were below the median wealth level than Batwa individuals (79.9%). In terms of sanitation, a greater percentage of Bakiga individuals had household washing facilities (47.4%) than the Batwa (33.9%). A greater percentage of Bakiga individuals had access to soap (52.0%) than Batwa individuals (35.1%) at the time of the survey.

AGI prevalence was greater among the Batwa adults than the Bakiga adults in the two survey periods that data were collected for both groups (July 2013 and April 2014) (Table 1). In July 2013, AGI prevalence was greater among the Batwa [9.70%, 95% confidence interval (CI) 5.93–13.47] than the Bakiga [4.48%, 95% CI 2.56–6.40]. In April 2014, AGI prevalence was also greater among the Batwa [2.94%, 95% CI 0.79–5.09] than the Bakiga [1.15%, 95% CI 0.03–2.27]. Prevalence estimates when a stricter international case definition was applied are presented in S1 File.

**Table 1:** Acute gastrointestinal illness (AGI) cases* for Batwa and Bakiga over 18 years old in southwestern Uganda (2013–2014).

	January 2013	July 2013		January 2014	April 2014	
	Batwa	Bakiga	Batwa	Batwa	Bakiga	Batwa
**Number of participants**	252	446	237	255	348	238
**Number of AGI cases**	11	20	23	12	4	7
**14-day AGI prevalence (%)**	4.37	4.48	9.70	4.71	1.15	2.94
**95% Confidence Interval**	1.85–6.89	2.56–6.40	5.93–13.47	2.11–7.31	0.03–2.27	0.79–5.09

*AGI was defined as self-reported vomiting and/or diarrhea in the previous 14 days, and excluded participants whose reported symptoms were due to chronic gastrointestinal illness, the use of medication, alcohol/ drugs, or pregnancy

Within each group, greater AGI prevalence was observed during survey periods following drier months than wetter months. Among the Bakiga, AGI prevalence was greater in the survey period following the driest month, July 2013 [prevalence 4.48%, June 2013 mean daily precipitation 0.24 mm], than in the survey period following the wettest month, April 2014 [prevalence 1.15%, March 2014 mean daily precipitation 5.09 mm]. Similarly, AGI prevalence was greatest among the Batwa in July 2013, also the survey period following the driest month (9.70%). Lower AGI prevalence was estimated in January 2013 (4.37%) and January 2014 (4.71%), the survey periods following months with intermediate precipitation [mean daily precipitation December 2012 3.76 mm, December 2013 3.36 mm]. Finally, the lowest AGI prevalence among the Batwa (2.94%) was also recorded following the wettest month, April 2014.

A multi-level multivariable logistic regression model of AGI occurrence, that included Indigenous identity and precipitation, and controlled for wealth and community-level effects indicated that Indigenous identity and decreased precipitation before a survey period were both significantly associated with increased odds of AGI (Table 2). The adjusted odds of AGI for Batwa were 1.91 times higher [odds ratio (OR) 1.91, 95% CI 1.12–3.26] than for Bakiga. For both Batwa and Bakiga, a one percent increase in log total precipitation in the 2 to 4 weeks before the survey lead to a decreased [OR 0.62 95% CI 0.49–0.79] decreased adjusted odds of AGI; that is, a 10 mm increase in total precipitation in the 2 to 4 weeks before survey results in a decreased odds ratio of AGI of 0.84.

**Table 2:** Multivariable multi-level logistic regression models with random intercepts to control for community-level clustering of AGI and associated factors in 10 communities.* Models are presented with Indigenous identity as a fixed effect as well as stratified by Indigenous identity**.

	Odds Ratio (95% Confidence Interval)		
	Both Bakiga and Batwa	Bakiga	Batwa
Log Total Precipitation (mm)***	0.62 (0.49, 0.79)	0.59 (0.39, 0.89)	0.64 (0.48, 0.87)
**Indigenous Identity**			
**Bakiga**	Ref		
**Batwa**	1.91 (1.12–3.26)		

*Clustering at the community level for the non-stratified model: full model (variance 0.07, 95% CI 0.00,1.06), Bakiga (variance 0.01, 95% CI 2.01, e-22- 4.78 e+17), Batwa (variance 0.27, 95% CI 0.00–30.07)

**Models controlled for wealth, a relative-asset based indicator of socio-economic identity.

***Log transformed total precipitation (mm) in the 2 to 4 weeks before each individual survey.

The results from the sensitivity analyses run with different precipitation lag periods are presented in S2 File. Also, additional potential independent variables, such as education, water source, water quality, water treatment, washing facilities, toilet type, animal exposure and animal ownership, were not included in the final model because they did not change the association of precipitation or Indigenous identity on AGI prevalence in bivariable and multivariable models (S3 File). Finally, models that had no-random effect structure, random effects for participants, and random effects for communities, had similar parameter estimates (S4 File).

**Fig 1:**
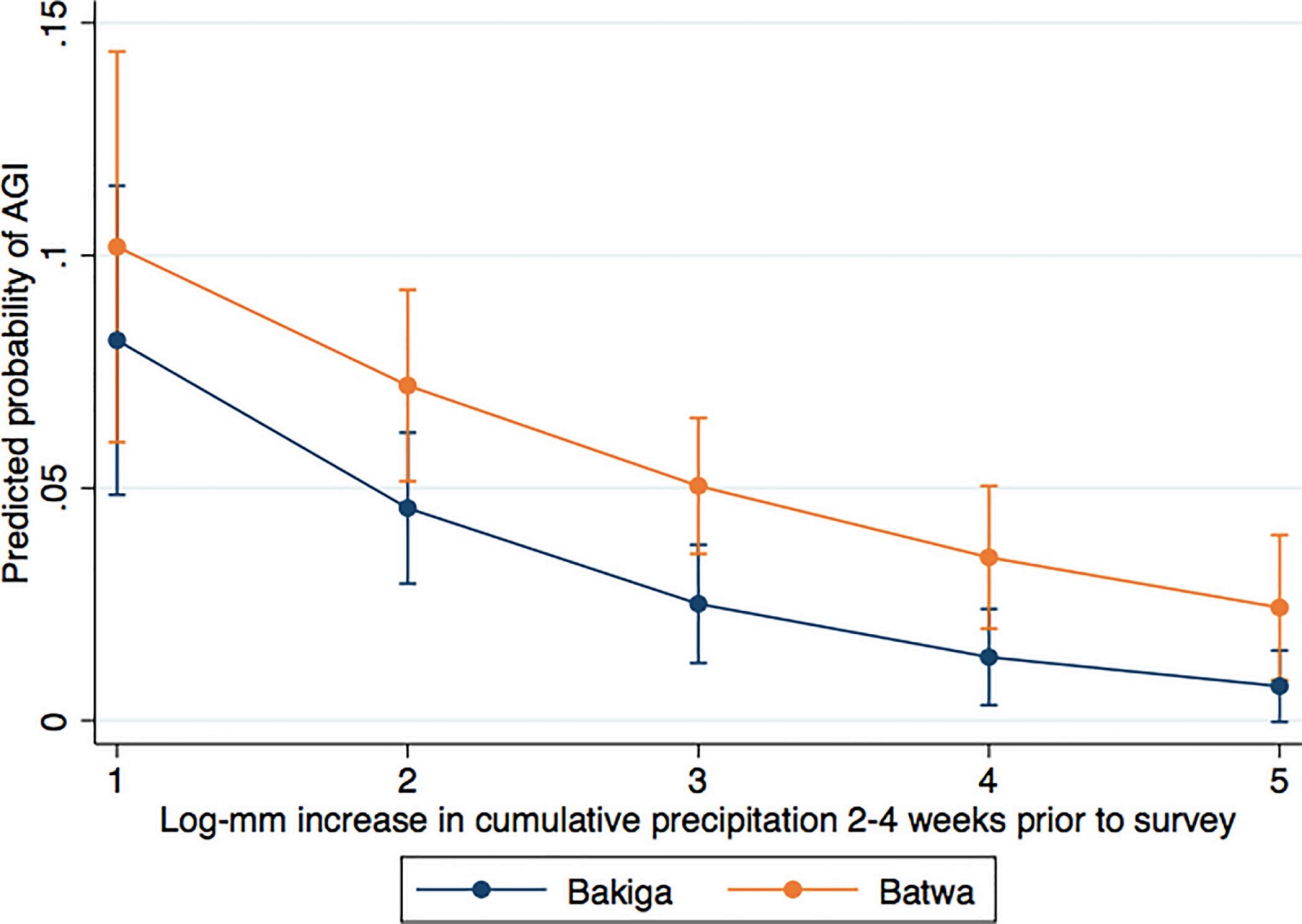
Marginal model predicted probability of AGI by log-mm increase in accumulated precipitation in the 2 to 4 weeks prior to survey for the Bakiga and Batwa in southwestern Uganda (2013–2014). Models included community-level random effects and controlled for wealth.

Indigenous identity did not modify the association between precipitation and AGI in our study. Models stratified by Indigenous identity indicated that increased precipitation in the weeks leading up to a survey had a significant and similarly protective effect on AGI occurrence among the Bakiga [OR 0.59, CI 0.39–0.89] and the Batwa [OR 0.61, CI 0.48–0.87] (Table 2). Though the Batwa and Bakiga had differences in baseline AGI prevalence (Batwa had greater odds of AGI than Bakiga). The model marginal predicted probabilities further highlight this relationship. As seen in Fig 1, as the accumulated log-mm precipitation in the two-four weeks prior to the survey increase, the predicted probability of AGI for both Batwa and Bakiga decrease with similar slopes, though the Batwa maintain a consistently higher baseline.

## Discussion

Our results support previous research, which has identified both Indigenous identity and decreased precipitation as risk factors for AGI in other countries [9–11]. Since we controlled for relative wealth in the multivariable models, our results suggest that Indigenous identity affects AGI occurrence beyond disparities in wealth. This finding is supported by a growing body of research demonstrating disparities in health outcomes between Indigenous populations and non-Indigenous populations [13]. In this context, differences in livelihoods or applicability of traditional knowledge may be driving the differing health outcomes, as the Batwa have recently transitioned from forest-based hunter-gather to agrarian lifestyles. The Batwa also face substantial social and political marginalization in Uganda, which often contributes to poor health outcomes [16, 23]. For instance, past research has shown that the Batwa are also at higher risk for malaria than the Bakiga, after controlling for wealth and other risk factors, and have less access to many health promoting preventative measures, such as insecticidal mosquito nets [28, 29].

Decreased precipitation in the weeks leading up to a survey period was also associated with greater odds of AGI for both Batwa and Bakiga. Previous studies of diarrheal disease in Africa similarly suggest that decreased precipitation is associated with increased diarrheal disease [9–11]. Decreased precipitation may increase risk of AGI through decreased water availability [39]. Decreased water availability can increase the concentration of pathogens in water sources [40], force people to rely on water sources of poorer quality [41], and modify hygiene practices, all of which can increase the risk of AGI [42–44].

Indigenous identity is thought to modify the effect that climate change has on infectious diseases, since Indigenous populations are often more vulnerable to climate change [23, 45]. However, our results suggest that, for southwestern Uganda, in the presence of decreased precipitation, AGI disparities between the two groups were maintained, rather than exacerbated. This suggests that, in this context, Indigenous identity does not modify the effect of precipitation on AGI occurrence and that these two factors both affect AGI occurrence independently. Therefore, the existing gradient of AGI prevalence between the two groups may be maintained rather than exacerbated as the future climate changes. Other studies investigated the effect modification of rainfall on diarrhea by other variables such as sex, age, socio-economic identity, social cohesion, and hygiene and sanitation practices, and found evidence of effect modification of these risk factors on diarrhea-precipitation association [46, 47]. Our results may challenge assumptions that current socially patterned disparities in AGI risk will necessarily be exacerbated by changing weather patterns caused by climate change.

The exclusion of children from the analysis is a limitation of our study. AGI research largely focuses on children, since they experience a higher burden of diarrheal disease than adults [6]. Future research should investigate whether precipitation and Indigenous identity impact children and adolescents differently than adults. Moreover, precipitation data for one community (Buhoma) was used for the entire study region of Kanungu District (1,274 km^2^), and it is possible that there are micro-climatic variations in precipitation which would reduce the accuracy of this independent variable. A more temporally robust understanding of the association between AGI and precipitation could have been gained by conducting AGI surveys across a greater number of time periods to be more representative of year-round precipitation-AGI dynamics. Survey data for the Bakiga population were restricted to July and April, but were not available for January surveys as for Batwa. This could have introduced bias in our comparative results. January experiences moderate precipitation, however, while July (dry) and April (wet) reflect the range of low and high precipitation throughout the year, minimizing the potential for bias across the full range of seasonal variation. Finally, similar to other studies that collect self-reported measures of illness, AGI occurrence estimates could be biased by respondent’s memory of illness (i.e., reporting bias), or by their interpretation or understanding of the question [48]. However, if reporting biases do exist in our data, it is unlikely that they differ across each survey period making temporal trends across surveys consistent. Additionally, our 14-day recall period to capture AGI episodes was deemed appropriate from pilot research among this population [23].

In conclusion, this study compared prevalence estimates between an Indigenous Batwa population and their non-Indigenous Bakiga neighbors in rural Uganda communities across survey periods with differing precipitation. Indigenous identity and decreased precipitation were both associated with increased odds of AGI. Though the Batwa experienced greater baseline AGI prevalence than the Bakiga, decreasing precipitation similarly increased the probability of AGI in both groups, maintaining this socially patterned health disparity rather than exacerbating or decreasing it. This suggests that, in this context, Indigenous status may not modify the effect of precipitation on AGI occurrence. These results may challenge the assumption that meteorological changes due to climate change will necessarily exacerbate health disparities along existing social gradients. Climate change may, in fact, maintain existing health gradients or create new ones depending on the geographical, cultural, social, and economic context.

## Supporting information

S1 File Prevalence estimates of AGI for international comparison. (DOCX)

S2 File Modelling the effect of different precipitation accumulation periods on AGI in full and stratified models. (DOCX)

S3 File Exploring potential confounding effects of covariates with bivariable logistic regression models of AGI occurrence. (DOCX)

S4 File Modelling the association between AGI occurrence, precipitation, and indigenous identity with no random-intercept structure, a fixed within-individual random intercept (temporal correlation structure which is exchangeable within each individual), and a fixed within-community random intercept (community-level correlation structure which is assumed to be exchangeable among individuals in the same community). (DOCX)
