# Supplementary material for: Is the effect of precipitation on acute gastrointestinal illness in southwestern Uganda different between Indigenous and non-Indigenous communities?
Source: PLoS One. 2019 May 2;14(5):e0214116. doi: 10.1371/journal.pone.0214116 (PMC6497252; doi:10.1371/journal.pone.0214116)
Supplement: S1 File — (DOCX) [file pone.0214116.s001.docx]

**Is the effect of precipitation on acute gastrointestinal illness in southwestern Uganda different between Indigenous and non-Indigenous communities?**

J Busch, L Berrang-Ford, S Clark, K Patterson, E Windfeld, B Donnelly, S Lwasa, D Namanya, IHACC team, S L Harper

**SI 1 Modelling the effect of different precipitation accumulation periods on AGI in full and stratified models**

Various precipitation (total mm prior to survey) accumulation periods were tested. Following testing, it was decided to include precipitation in the 2 to 4 weeks before each survey into the final model. The relationship between precipitation and AGI was similar regardless of whether the accumulation period was 2 to 4, 2 to 5, 2 to 6, 2 to 7, or 2 to 8 weeks before each survey (Table S1).

**Table S1: Multivariable mixed-effects logistic regression models (full and stratified by indigenous status) of the effect of precipitation accumulation period exposures on AGI occurrence in 10 Indigenous and non-indigenous communities in rural southwestern Uganda.** Models included community-level random intercepts and controlled for wealth (asset-based indictor) as a fixed-effect.

|  |  | Full model | Bakiga model | Batwa model |
| --- | --- | --- | --- | --- |
|  |  | **Adjusted Odds Ratio (95% Confidence Interval)** | | |
| Total 3-week precipitation (accumulated 2 to 5 weeks prior to the survey) | Log Total Precipitation (mm)* | 0.58 (0.45-0.76) | 0.64 (0.45-0.91) | 0.53 (0.36-0.77) |
|  | Indigenous Status |  |  |  |
|  | Bakiga | ref |  |  |
|  | Batwa | 2.79 (1.52-5.13) |  |  |
| Total 4-week precipitation (accumulated 2 to 6 weeks prior to the survey) | Log Total Precipitation (mm)* | 0.56 (0.41-0.76) | 0.60 (0.40-0.90) | 0.52 (0.33-0.82) |
|  | Indigenous Status |  |  |  |
|  | Bakiga | ref |  |  |
|  | Batwa | 2.76 (1.49-5.12) |  |  |
| Total 5-week precipitation (accumulated 2 to 7 weeks prior to the survey) | Log Total Precipitation (mm)* | 0.57 (0.43-0.76) | 0.61 (0.41-0.90) | 0.54 (0.37-0.80) |
|  | Indigenous Status |  |  |  |
|  | Bakiga | ref |  |  |
|  | Batwa | 3.04 (1.62-5.70) |  |  |
| Total 6-week precipitation (accumulated 2 to 8 weeks prior to the survey) | Log Total Precipitation (mm)* | 0.53 (0.39-0.72) | 0.60 (0.39-0.93) | 0.47 (0.30-0.72) |
|  | Indigenous Status |  |  |  |
|  | Bakiga | ref |  |  |
|  | Batwa | 3.11 (1.66-5.84) |  |  |
