# Supplementary material for: Is the effect of precipitation on acute gastrointestinal illness in southwestern Uganda different between Indigenous and non-Indigenous communities?
Source: PLoS One. 2019 May 2;14(5):e0214116. doi: 10.1371/journal.pone.0214116 (PMC6497252; doi:10.1371/journal.pone.0214116)
Supplement: S2 File — (DOCX) [file pone.0214116.s002.docx]

**Is the effect of precipitation on acute gastrointestinal illness in southwestern Uganda different between Indigenous and non-Indigenous communities?**

J Busch, L Berrang-Ford, S Clark, K Patterson, E Windfeld, B Donnelly, S Lwasa, D Namanya, IHACC team, S L Harper

**S2 Exploring potential confounding effects of covariates with bivariable logistic regression models of AGI occurrence**

The potential associations between AGI occurrence (outcome variable), indigenous identify (independent variable) and exposure to precipitation (independent variable) are the primary associations of interest in this paper. To control for potential confounding effects this these models, we explored the potential associations between AGI and independent variables determined *a priori* as potential confounders through a series of bivariable logistic regression models. Potential confounders with a p-value <0.25 were considered for inclusion in the full models of AGI,

**S2 Table 1: Univariable logistic regression models (AGI and one potential risk factor).**

| Variable | Categories | Odds Ratio | 95% Confidence Interval | | P-value |
| --- | --- | --- | --- | --- | --- |
| Demographic Variables: |  |  |  |  |  |
| Indigenous Status | Bakiga | ref |  |  |  |
|  | Batwa | 1.83 | 1.12 | 2.99 | 0.016 |
| Sex | Male | ref |  |  |  |
|  | Female | 0.93 | 0.58 | 1.48 | 0.758 |
| Wealth  (2 categories) | Below Median | ref |  |  |  |
|  | Above Median | 0.73 | 0.44 | 1.21 | 0.214 |
| Employment | None | ref |  |  |  |
|  | Paid | 1.65 | 0.86 | 3.17 | 0.133 |
|  | Self-employed | 0.78 | 0.42 | 1.46 | 0.443 |
|  | Non-paid employed | 5.77 | 1.58 | 21.13 | 0.008 |
| Education | No school | ref |  |  |  |
|  | Some primary | 1.00 | 0.56 | 1.80 | 0.999 |
|  | Primary and higher | 0.64 | 0.19 | 2.23 | 0.487 |
| Community * | 1 | ref |  |  |  |
|  | 2 | 1.69 | 0.40 | 7.19 | 0.475 |
|  | 3 | 4.26 | 1.17 | 15.55 | 0.028 |
|  | 4 | 2.79 | 0.74 | 10.46 | 0.128 |
|  | 5 | 3.32 | 0.88 | 12.47 | 0.075 |
|  | 6 | 3.80 | 1.05 | 13.67 | 0.041 |
|  | 7 | 4.49 | 1.23 | 16.37 | 0.023 |
|  | 8 | 1.32 | 0.29 | 5.98 | 0.719 |
|  | 9 | 3.88 | 1.01 | 14.89 | 0.049 |
|  | 10 | 2.56 | 0.60 | 10.92 | 0.203 |
| AGI Risk Factors: |  |  |  |  |  |
| Washing Facilities | No | ref |  |  |  |
|  | Yes | 0.88 | 0.54 | 1.42 | 0.592 |
| Own Animals | No | ref |  |  |  |
|  | Yes | 0.78 | 0.47 | 1.28 | 0.327 |
| Boil Water | No | ref |  |  |  |
|  | Yes | 0.78 | 0.48 | 1.24 | 0.291 |
| Treat Water | No | ref |  |  |  |
|  | Yes | 0.83 | 0.52 | 1.33 | 0.439 |
| Toilet Type | Other | ref |  |  |  |
|  | Covered | 0.71 | 0.42 | 1.23 | 0.223 |

*Community names removed to protect anonymity of participants
