# Supplementary material for: Is the effect of precipitation on acute gastrointestinal illness in southwestern Uganda different between Indigenous and non-Indigenous communities?
Source: PLoS One. 2019 May 2;14(5):e0214116. doi: 10.1371/journal.pone.0214116 (PMC6497252; doi:10.1371/journal.pone.0214116)
Supplement: S3 File — (DOCX) [file pone.0214116.s003.docx]

**Is the effect of precipitation on acute gastrointestinal illness in southwestern Uganda different between Indigenous and non-Indigenous communities?**

J Busch, L Berrang-Ford, S Clark, K Patterson, E Windfeld, B Donnelly, S Lwasa, D Namanya, IHACC team, S L Harper

**S3 Prevalence Estimates of AGI for International Comparison**

Due to low case numbers, a more inclusive case definition was used for analysis in this study. Prevalence estimates used a stricter case definition proposed by Majowicz et al. (2008) (3 or more loose stools or any vomiting) are provided here.

**Table S3: AGI cases (reported vomiting or diarrhea in the previous 14 days) for Batwa and Bakiga over 18 years old, excluding pregnant women using the case definition proposed by Majowicz et al. (2008) (3 or more loose stools or any vomiting).**

|  | January 2013 | July 2013 | | January 2014 | April 2014 | |
| --- | --- | --- | --- | --- | --- | --- |
|  | **Batwa** | **Bakiga** | **Batwa** | **Batwa** | **Bakiga** | **Batwa** |
| **n** | 252 | 446 | 237 | 255 | 348 | 238 |
| **AGI Cases (Majowicz Definition)** | 11 | 14 | 20 | 10 | 4 | 6 |
| **14-day Prevalence (%)** | 4.37 | 3.14 | 8.33 | 3.92 | 1.15 | 2.42 |
| **95% Confidence Interval** | +/-2.52 | +/- 1.62 | +/- 3.50 | +/- 2.38 | +/- 1.12 | +/- 1.99 |

**Works Cited**

Majowicz, S. E., Hall, G., Scallan, E., Adak, G., Gauci, C., Jones, T., . . . Sockett, P. (2008). A common, symptom-based case definition for gastroenteritis. *Epidemiology and Infection, 136*(07), 886-894.
