# Supplementary material for: Is the effect of precipitation on acute gastrointestinal illness in southwestern Uganda different between Indigenous and non-Indigenous communities?
Source: PLoS One. 2019 May 2;14(5):e0214116. doi: 10.1371/journal.pone.0214116 (PMC6497252; doi:10.1371/journal.pone.0214116)
Supplement: S4 File — (DOCX) [file pone.0214116.s004.docx]

**Is the effect of precipitation on acute gastrointestinal illness in southwestern Uganda different between Indigenous and non-Indigenous communities?**

J Busch, L Berrang-Ford, S Clark, K Patterson, E Windfeld, B Donnelly, S Lwasa, D Namanya, IHACC team, S L Harper

**S4 Modelling the association between AGI occurrence, precipitation, and indigenous identity with no random-intercept structure, a fixed within-individual random intercept (temporal correlation structure which is exchangeable within each individual), and a fixed within-community random intercept (community-level correlation structure which is assumed to be exchangeable among individuals in the same community).**

**S4 Table 1. Multivariable standard (a) mixed-effects (b, c) logistic regression models of the effect of 2- to 4-week precipitation accumulation exposure on AGI occurrence in 10 Indigenous and non-indigenous communities in rural southwestern Uganda.** Models controlled for wealth (asset-based indictor) and indigenous identify as a fixed-effects.

|  | 1. **No Random Effect** | 1. **Individual as**   **Random Effect** | 1. **Community as Random Effect** |
| --- | --- | --- | --- |
|  | Odds Ratio (95% Confidence Interval) | | |
| Log Total Precipitation (mm)* | 0.63 (0.49-0.79) | 0.62 (0.49-0.79) | 0.62 (0.49-0.79) |
| Indigenous Status |  |  |  |
| Bakiga | ref | ref | ref |
| Batwa | 1.92 (1.13-3.26) | 1.94 (1.12-3.35) | 1.91 (1.12-3.27) |
| AIC | 617.03 | 618. 93 | 618.31 |
| BIC | 638.91 | 646.28 | 645.67 |

*Log transformed total precipitation in the 2-4 weeks before the survey.

**Models controlled for wealth, a relative-asset based indicator of socio-economic status.

Model fit was compared through AIC and BIC values.
